# Effective Conditions for Achieving Carbon Unlocking Targets for Transport Infrastructure Development—Joint Analysis Based on PLS-SEM and NCA

**DOI:** 10.3390/ijerph20021170

**Published:** 2023-01-09

**Authors:** Yun Chen, Da Wang, Wenxi Zhu, Yunfei Hou, Dingli Liu, Chongsen Ma, Tian Li, Yuan Yuan

**Affiliations:** 1 School of Traffic & Transportation Engineering, Changsha University of Science and Technology, Changsha 410000, China; 2 National Engineering Research Center of Highway Maintenance Technology, Changsha University of Science & Technology, Changsha 410114, China

**Keywords:** carbon unlocking, transport infrastructure, PLS-SEM, necessary condition analysis, SDGs

## Abstract

It is important to investigate how to achieve carbon unlocking in the transport sector, especially in transport infrastructure, in order to contribute to the achievement of carbon neutrality targets and the 2030 Sustainable Development Goals. This study aims to investigate the necessary and sufficient conditions to achieve carbon unlocking in transport infrastructure. To achieve this, a combination of partial least squares structural equation modeling (PLS-SEM) and necessary condition analysis (NCA) methods have been used to examine whether there are unidentified necessity factors beyond the currently recognized ‘technology-in-institution’ (TIC) lock-in. This study also explores how the carbon unlocking of transport infrastructure can be achieved through the unlocking of relevant factors. The study includes 366 points from a subjective questionnaire from the government, transport infrastructure researchers, and relevant businesspeople. We found that, at the adequacy level, achieving institutional and technological unlocking is sufficient and economic factors have little impact on transport infrastructure (0.06), and that institutional and technical factors have a large impact on carbon unlocking (0.453, 0.280); however, from the necessary point of view, carbon unlocking at the economic level is necessary to achieve the goal of a medium to high level of carbon unlocking. To achieve carbon unlocking at this level (over 50%), a combination of technological, institutional, and economic factors is required. To achieve full carbon unlocking, the technology, system, and economy need to be at least 0.533, 0.791, and 0.63 unlocked. Therefore, we can conclude that by using the joint analysis of PLS-SEM and NCA, we have achieved an extension of the traditional TIC and identified sufficient and necessary conditions to achieve a medium to high degree of carbon unlocking.

## 1. Introduction

**Figure 1:**
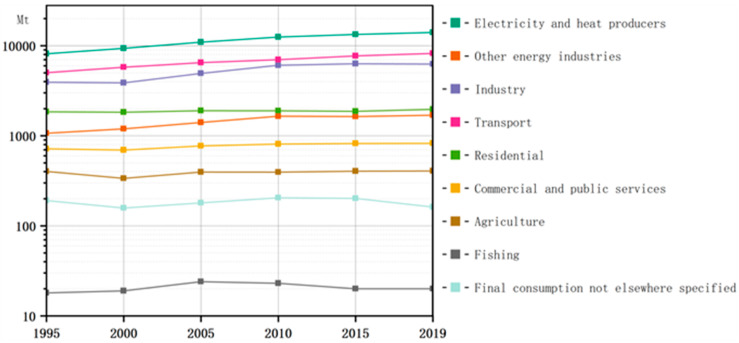
${\mathrm{CO}}_{2}$ emission table by sector.

Rapid temperature change has become an important environmental issue worldwide and poses a major threat to human survival and development. Most scholars believe that the rapid increase in ${\mathrm{CO}}_{2}\;$ emissions resulting from high-carbon development patterns is the main cause of temperature rise. Therefore, the international community has adopted various measures to control ${\mathrm{CO}}_{2}$ emissions. However, some experts have pointed out that the control of ${\mathrm{CO}}_{2}$ emissions at a technical level alone cannot promote the transformation of society as a whole towards low-carbon, efficient, and sustainable development. To fundamentally reduce ${\mathrm{CO}}_{2}$ emissions, the current high-carbon development model needs to be changed, and industries need to be “unlocked” from the high-carbon development model to facilitate the implementation of a low-carbon economy. Currently, industry, energy and power, and transport are important sources of ${\mathrm{CO}}_{2}$ emissions (see Figure 1) [1].

Meanwhile, as the economy grows and communication between regions increases, the demand for transport infrastructure and means of transport will also increase, leading to a further increase in the carbon footprint of the transport system. This calls for governments, industry, and researchers to find a way to move transport away from a high-carbon development model. Targeting transport system improvements is an important way to achieve the goals of the United Nations 2030 Agenda for Sustainable Development Goals (SDGs). Wenbin et al. point out that buildings and infrastructure have a significant impact on the environment and that this can be achieved in a number of ways, but most research has focused on how to reduce the carbon emissions of transport and on sustainable development, and less attention has been paid to transport infrastructure itself [2]. At the same time, SDG9 calls for resilient infrastructure that promotes inclusive and sustainable industrialization. However, the current transport infrastructure, locked in a high-carbon development pathway by both institutions and technology, has raised the level of difficulty in achieving low-carbon sustainable development in transport infrastructure. However, in order to achieve the objectives of the SDGs, it is necessary to explore how to achieve carbon unlocking in transport infrastructure development. In addition, in order to improve the effectiveness and reliability of the measures, it is also necessary to explore the conditions necessary to achieve carbon unlocking in transport infrastructure, as well as those conditions considered sufficient to achieve carbon unlocking in transport infrastructure. In this way, policymakers and business managers can identify the key factors that influence the outcome and how to improve the control path when recommending carbon unlocking for transport infrastructure.

Most studies on transport infrastructure and carbon unlocking have focused on the carbon emissions of transport infrastructure and the impact of transport infrastructure on carbon emissions, while fewer scholars have focused on how to promote the decoupling of transport infrastructure and related industries from high-carbon development patterns at the economic, institutional and technological levels [3].

In order to identify the necessary factors and sufficient conditions for achieving carbon unlocking in transport infrastructure, we combine partial least squares structural equation modeling (PLS-SEM) with necessary conditions analysis (NCA) to analyze the problem. The contributions of this study focus on two main points.

(1) The existing studies on carbon locking and unlocking pathways are mostly focused on the energy and power sectors [4,5,6,7]. Most of the studies in the field of transportation also focus on transportation vehicles, but less on transportation infrastructure [3,8,9,10]. In the research process, scholars also focus on the key factors that lead to carbon locking and unlocking, but less on the factors that are necessary but less influential in the locking and unlocking process [2,8,11,12]. This research focuses on whether there are factors other than the traditional technical, institutional factors that are responsible for carbon locking in the transportation infrastructure sector. By extending the traditional PLS-SEM method, this study introduces the NCA analysis method to explore whether there are factors that have less influence but are necessary in the carbon unlocking process. This research introduces economic factors into the analysis of carbon locking in transport infrastructure, and analyzes the factors and processes affecting carbon unlocking in transport infrastructure, so as to enhance the possibility of achieving the SDG and carbon neutrality targets. The current research found that economic factors, although playing a smaller role in the carbon unlocking process, must be involved if a medium to high level of carbon unlocking is to be achieved.

(2) Key bottlenecks affecting carbon unlocking are identified, and effective strategies to improve the likelihood of achieving carbon unlocking targets and efficiency based on the analysis results are discussed.

At the structural level of this paper, the second part will provide a review of the theoretical background of the study and related literature, and analyze the techno-economic lock-in factors and their applicability in the field of transport infrastructure; the third part will establish research hypotheses based on existing literature and research results; the fourth part will introduce our research methodology and procedures; the fifth and sixth parts will use PLS-SEM and NCA to analyze the sufficient and necessary conditions affecting carbon unlocking in transport infrastructure; finally, the results will be discussed and policy recommendations will be provided.

## 2. Literature Review and Research Hypothesis

This section reviews the extensive literature on carbon locking and carbon unlocking, identifies the drivers common to previous studies for achieving carbon unlocking, innovatively explores the impact of economic factors on carbon unlocking, proposes a major hypothesis, and applies some supporting assumptions as a basis for PLS-SEM and NCA analysis.

### 2.1. Main Hypothesis

Numerous scholars are paying close attention to the unlocking of carbon lock-in to reconcile the economy with climate change, especially in some carbon-intensive areas, such as transportation infrastructure development. The general opinion is that carbon lock-in refers to the tendency of certain carbon-intensive technology systems to persist over time. With the positive feedback of increasing returns to scale, carbon-based technologies and systems evolve synergistically to form a “technology–institution complex” (TIC), which can be broadly understood as a socio-technical system or infrastructure system [13,14,15,16]. In other words, the causes of carbon lock-in focus on the interaction between technology and institutions; thus, carbon lock-in can be subdivided into technology lock-in and institutional lock-in. Institutional lock-in differs from technology lock-in in terms of intentional characteristics, institutional nature, and differences in political processes and markets [4]. However, if the roles of technology and institutions reinforce each other, industrialized countries suffer from carbon lock-in due to their highly evolved TICs, making it very difficult to escape from high-carbon mobility systems [17]. In addition, those countries also experience persistent market and policy failures [18].

It is not hard to find existing studies which have mainly highlighted the role of technology, institutions, and the technology–institution complex, ignoring that the growth of economies of scale can also greatly inhibit the innovation and competitiveness of low-carbon alternatives, which can have a huge impact on carbon lock-in. Therefore, in order to determine that economic factors play an important role in achieving the carbon unlocking goal for transportation infrastructure, a major hypothesis is proposed in this paper, which is as follows.

**H1.**  *Technical factors can drive the realization of carbon unlocking*.

**H2.**  *Institutional factors can drive the realization of carbon unlocking*.

**H3.**  *Economic factors can drive the realization of carbon unlocking*.

### 2.2. Support Hypothesis

In the main hypothesis of this paper, three types of factors including technological, institutional, and economic, work together to help get out of the carbon lock-in dilemma. However, these factors are macro-level conceptual aggregates which are not easily understood. In order to gain a deeper understanding of the specific forms in which these three types of drivers influence carbon unlocking, some supporting hypotheses are proposed separately to form a pathway to break carbon locking.

#### 2.2.1. Technical Factors

Technological power is an integral part of the carbon unlocking process [19]. There are case studies suggesting that resources from several sectors can strengthen the development of sustainable technologies and initiate pathways to a low-carbon economy [20]. Existing technologies benefit from increasing returns over time, leading to technology lock-in and path dependence, which hinder the development of new technologies. Those new technologies reduce greenhouse gases, improve resource use, and increase energy efficiency, and can be specifically classified as high-carbon alternatives, energy Internet technologies, and digital regulation technologies.

The substitution or decarbonization transition of carbon-based technology regimes can help move away from carbon lock-in through its role, development, deployment, and proliferation [21]. First, it is important to clearly realize the role of carbon-based alternative technologies. Obviously, existing conventional technologies are insufficient to achieve the dual carbon goal alone; this is because the adoption of low-carbon technologies does not automatically eliminate high-carbon technologies [22]. Technology alternative routes seem to be inevitable and connected [23,24,25,26,27]. However, since path dependency based on incremental benefits exists for all forms of transportation infrastructure, future development paths will continue this lock-in if measures to break the carbon lock-in are not implemented [28]. Therefore, there is a need to break the path dependence in the direction of technology development. Even so, the transition to low-carbon technologies in high-carbon sectors is still a challenge [6,29]. These newly developed technologies have high unit costs as they have not yet benefited from economies of scale, learning effects, adaptive expectations, and network effects [30]. Reducing the difficulty of trading or transferring low-carbon technologies would be a powerful measure to address this issue. Additionally, a comparison of alternative energy patents with other types of patents reveals that alternative technologies are cited more frequently and have a wider range of applications, which indicates their higher social value [31]. Low-carbon technologies are used more frequently to promote decarbonization if they are widely disseminated.

Energy Internet technologies also play an important role in carbon unlocking. Rapid deployment of technology generates experience that feeds into technology improvements [32]. Energy Internet technology interconnects energy nodes such as electricity, oil, and natural gas, allowing each individual energy consumption and carbon emission indicator to be measured with digital precision, and various energy-consuming needs to be supplied with the most efficient production. This distributed technology can be used to reduce overall infrastructure demand and reduce carbon emissions [33].

Digital regulatory technology is considered to be one of the technological factors affecting carbon unlocking. There is a significant positive relationship between regulatory compliance spending and R&D spending in regulated industries, although the magnitude of the effect is not significant [34,35]. Regulators can improve their efficiency and strengthen the supervision of carbon locking practices through digital regulatory technology.

Based on the above discussion, the following hypotheses are then proposed:

**H1a.**  *The development of replacements for high-carbon technologies can promote the realization of carbon unlocking*.

**H1b.**  *The promotion difficulty in the application of low-carbon technologies can push the realization of carbon unlocking*.

**H1c.**  *Cracking the path dependency of technology R&D direction can drive the realization of carbon unlocking*.

**H1d.**  *Reducing the difficulty of low-carbon technology trading or transfer promotes carbon unlocking*.

**H1e.**  *The application of energy Internet technology in the carbon unlocking process will help to promote carbon unlocking*.

**H1f.**  *The application of digital regulatory technologies contributes to the achievement of carbon unlocking goals*.

#### 2.2.2. Institutional Factors

Institutional factors associated with carbon-based technologies are an important cause of carbon lock-in in transportation infrastructure, and therefore, an important breakthrough to achieve carbon unlocking [7,36]. Scholars who hold the view of institutional unlocking argue that technology lock-in is a superficial result, and that carbon lock-in must be fully formed by institutional reinforcement, so the most promising way to achieve carbon unlocking is to promote institutional lock-in of new decarbonization trajectories [4].

Stopping collusion between government and business can appropriately reduce the probability of carbon lock-in occurring. Institutional lock-in is often manifested in alliances and power distribution among players that support the continued dominance of fossil fuels [37]. For example, regional governments play the role of policy makers and represent the general public’s interests on the one hand, and are subject to performance reviews by their superiors on the other. Companies need to implement the government’s requirements but seek a certain level of profit. Under this circumstance, stakeholders seek to stabilize their own power or strengthen the institutions that help them achieve their interests, rather than promote fundamental change. Thus, reducing collusion among related stakeholders and encouraging cooperative unlocking is needed [8,38].

Implementation of low-carbon system helps escape the carbon lock-in dilemma. It is thought to be an open system all stakeholders need to actively participate in. The degree of emphasis on the “openness” of policy development and the vision of policy outcomes determines the unlocking effect [39].

The inertia of the development model needs to be broken at the institutional level in parallel [40]. Large-scale institutional policy change often requires external shocks or “extraordinary events” [41,42]. So-called windows of opportunity are opened in response to external shocks and are then used by policy entrepreneurs to drive policy change [43]. In addition, institutional level transformation may help to break the technological stranglehold [44].

The adoption of incentives that favor the development of low-carbon technologies would minimize lock-in with traditional technologies [45,46]. Permanent policy interventions, such as financial subsidies and taxes, are needed if structural change from carbon-intensive to low-carbon production is to be achieved [47,48,49]. Government plays an important role through its policies [17]. Appropriate financial subsidies from the government will reduce the cost of enterprises and further encourage their low-carbon technology innovation.

It is important to note that carbon unlocking through policy interventions is uniquely challenging compared to technical or behavioral lock-in mechanisms, because institutional policies have the ability to override market forces. Radical reforms and reconfigurations of the energy system can even carry the risk of backlash, collapse, or lock-in [50]. To prevent such a situation, digital control models are necessary.

Therefore, we have the following assumptions.

**H2a.**  *Reducing the level of collusion between regional governments and enterprises can contribute to the achievement of carbon unlocking targets*.

**H2b.**  *Improving the implementation of low-carbon systems can promote the realization of carbon unlocking targets*.

**H2c.**  *Breaking the inertia of the traditional high-carbon development model at the institutional level can promote the achievement of the carbon unlocking goal*.

**H2d.**  *Improving financial subsidies for low-carbon technologies can promote the realization of the carbon unlocking goal*.

**H2e.**  *Constructing low-carbon digital control models can promote the realization of carbon unlocking targets*.

#### 2.2.3. Economic Factors

In the economic dimension, carbon unlocking of transportation infrastructure can be achieved in three ways, namely, adjusting the industrial structure of transportation infrastructure, increasing the proportion of investment in technology research and development, and strengthening the linkage between the low-carbon development model and the regional economy.

Primarily, industrial structure and economic scale are critical contributors to regional carbon decoupling [12,51]. The expansion of the share of clean energy and the digital economy are effective approaches to optimize the industrial structure [52]. Industrial structure optimization, along with energy efficiency improvement and energy structure upgrading, are the three main types of government interventions to achieve emission reduction targets [53]. The industrial structure reflects the complex relationships or connectivity between different industrial sectors and how each industry affects the overall industrial network. Structural optimization is aimed at changing the proportion of industrial sectors in order to reduce total emissions and energy consumption [54]. The effects of industrial structure on carbon emissions have been confirmed in many studies, and a large number of scholars believe that industrial restructuring has a positive impact on mitigating carbon emissions [55,56,57,58]. The critical point to achieve emission reduction through industrial restructuring is to identify and control the emission-dominant part, rather than the economically leading part [59].

Secondly, adjustment of investment patterns becomes a key economic factor for carbon decoupling, given the crucial positive role of fixed asset investment in carbon emission growth [11,60]. Global infrastructure investment is at an all-time high, so more and more decisions are now being made that will lock in development patterns for future generations [61]. Accounting for the presence of crowding-out effects, investments in low-carbon technologies are found to crowd out other non-emission reducing investments in the plant [62,63]. Thus, investments in corporate R&D of low-carbon technologies can provoke a range of environmental innovations and reduce dependence on natural resources by achieving more efficient technologies, rather than those promoting economic growth but causing higher energy consumption [64,65,66]. Simply investing more in infrastructure itself is highly likely to exacerbate locking [67].

Additionally, implementing low-carbon strategies through regional integration will contribute to decoupling economic growth from carbon as well [68]. The transition to low-carbon development in regional economies is a complex process, and there are large differences and complementarities in the development of low-carbon economies in regions with different levels of development [69]. Changes in the percentage of the low-carbon economy in regional economic growth are determined by many factors, such as the density of land development [70]. Some scholars have developed financial tools such as low-carbon financial indices, which score as an indication of a country’s investment in low-carbon technologies to maximize the share of output and R&D in the future [9,71,72]. Promoting the establishment of carbon trading markets is a powerful tool to link low-\carbon development models to regional economies, reducing carbon emissions through market mechanisms, with similar incentives for instruments such as environmental regulatory technologies and market-based environmental taxes [12,73,74,75].

With these observations, the following hypotheses are formulated:

**H3a.**  *Adjusting the industrial structure of transportation infrastructure to enable the low-carbon development model at a high profit level contributes to the achievement of the carbon unlocking target*.

**H3b.**  *Increasing the proportion of enterprises’ R&D investment in low-carbon technology facilitates the achievement of the carbon unlocking target*.

**H3c.**  *Promoting the linkage between the low-carbon development model and regional economic growth is helpful to promote the achievement of the carbon unlocking target*.

Due to the large amount of literature involved, we summarized the research perspectives of some scholars and explored some shortcomings of research methods they applied to better carry out the research of this thesis, as shown in Table 1.

## 3. Methods and Materials

### 3.1. Sample

The survey population for this study included people from the government, transport infrastructure research scholars, and large transport construction groups. A total of 500 questionnaires were distributed and 366 valid questionnaires were returned, with an effective rate of 73.2%. The questionnaire distribution process was mainly completed through online distribution and in-person interviews. Among the surveyed groups, 52.7% were male and 47.3% were female; the number of years of work and types of work of the surveyed groups are shown in Table 2. The distribution of the surveyed population is in line with the actual situation and analysis needs, and further analysis and research can be conducted.

### 3.2. Measures

The main body of the questionnaire consisted of several sub-sections containing questions related to the factors influencing the carbon unlocking of transport infrastructure, and also targeted the respondent group on the impact of multiple factors acting together on carbon unlocking. The questionnaire builds on existing research by classifying the factors influencing carbon unlocking in transport infrastructure into three different dimensions, namely, technical, institutional, and economic factors (see Table 3). These factors were formulated as statements and measured on a five-point Likert scale. The degree of carbon unlocking was judged by the respondent, based on the empirical judgment of the impact that the combination of the above factors may have on the carbon unlocking of transport infrastructure.

### 3.3. Data Analysis Method Selection

The correct method must be used to identify the necessary and sufficient conditions that influence the carbon unlocking of transport infrastructure. Traditional SEM methods usually ignore certain pathways that are less influential, but decisive or critical for achieving the final goal. Therefore, we have added the Necessary Conditions Analysis (NCA) method to the traditional SEM method for necessity analysis.

NCA is a data analysis technique for identifying necessary (but not sufficient) conditions in a data set. It implements a refinement and complement to traditional regression-based data analysis. The method was originally developed by Dul et al. and was then used by Richter, Schubring, and others in several research areas [76]. Richter points out through his research that PLS-SEM and NCA enable researchers to explore and validate hypotheses following a sufficiency logic, as well as hypotheses drawing on a necessity logic [77,78].

In the selection of the SEM method, we followed the findings of Hair and Rigdon et al. and chose PLS-SEM as the method of analysis based on a full comparison of CB-SEM and PLS-SEM, and based on the characteristics of the research topic and the quality of the sample. The research process in this paper refers to the research methods and steps of Alexandre, Richter, and Hair et al. The study first calculates the weight of each indicator and the model quality evaluation index through PLS-SEM and saves the potential variable scores (non-standardized) calculated by PLS-SEM as the input of NCA. That is, the PLS-SEM method is used to identify the factors that play an important role in the carbon unlocking process of transport infrastructure, and on this basis, the NCA is used to identify the critical factors that lead to the achievement of the carbon unlocking target of transport infrastructure [10,78,79].

## 4. PLS-SEM Result

When using PLS-SEM for analysis, the potential variables and structural models need to be evaluated first, according to Hair et al., Liu et al. [80,81], and the PLS-SEM manual. We used SmartPLS v4 to analyze the data.

### 4.1. Measurement Models

Before the relationship between the composites and indicators of the model can be analyzed, the reliability and validity of the model needs to be evaluated first. The mean and standard deviation of the responses to each question of the questionnaire are shown in Table 4. The indicators analyzed are shown in Table 5. In this study, the indicator loadings, internal consistency reliability (CR), average variance extracted (AVE), and discriminant validity (HTMT) were selected for analysis with reference to Wenbin et al. [2]. The Goodness-of-fit is shown in Table 6. The heterotrait–monotrait ratio is shown in Table 7.

Examples of suggested fits in the PLS-SEM model include the standardized root mean square residual (SRMR) and GOF. In PLS-SEM models, meanwhile, $R^{2}$ (predictive effect value) and $Q^{2}$ (predictive correlation) are commonly used in PLS-SEM models to evaluate the predictive power of the model. In PLS-SEM, with $R^{2}$ greater than 0.5, we consider that the explanatory power of the model meets the requirements; the $R^{2}$ in this study is 0.561, which basically meets the requirements. The model’s $Q^{2}$ is 0.553, which also meets the requirement.

Existing researchers argue that the traditional CB-SEM model fit evaluation metrics are not applicable to PLS-SEM. According to Shmueli et al. and Chin et al., metrics such as HTMT, CR, and SRMR are usually chosen to evaluate models in PLS-SEM [82,83]. However, it should be noted that the goodness-of-fit criteria in PLS-SEM do not represent a valid measure of model fit [80].

The calculations show that all CRs in the constructed model are greater than 0.899; therefore, the model has a high degree of internal inconsistency. Almost all the Factor Loading was greater than 0.727 and the data also had convergent validity. In addition, according to the goodness-of-fit (GOF) formula, if the GOF value is greater than 0.26, the model is considered to have good applicability in the field of humanities and social sciences. In this study, GOF = 0.607, indicating that the model fits well.

According to Table 6, we can find that all the indicators of the model meet the requirements and can be further analyzed.

In addition, the discriminant validity measured by the heterogeneity–monogeneity (HTMT) ratio was confirmed, as all measures were below the threshold of 0.85 [10]. Therefore, we can conclude that the constructed measurement model is compliant.

### 4.2. Structural Model Assessment

**Figure 2:**
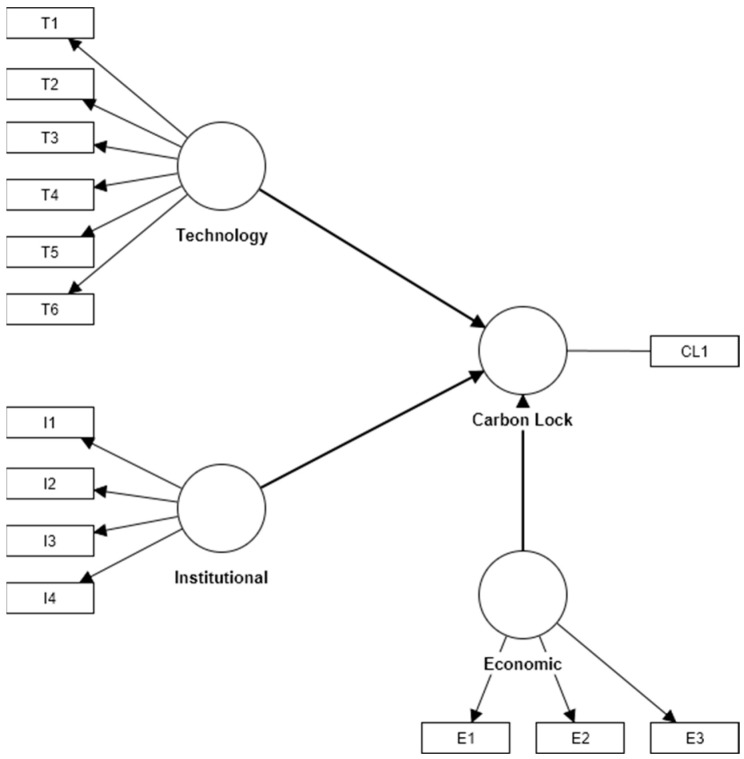
Structural model.

**Figure 3:**
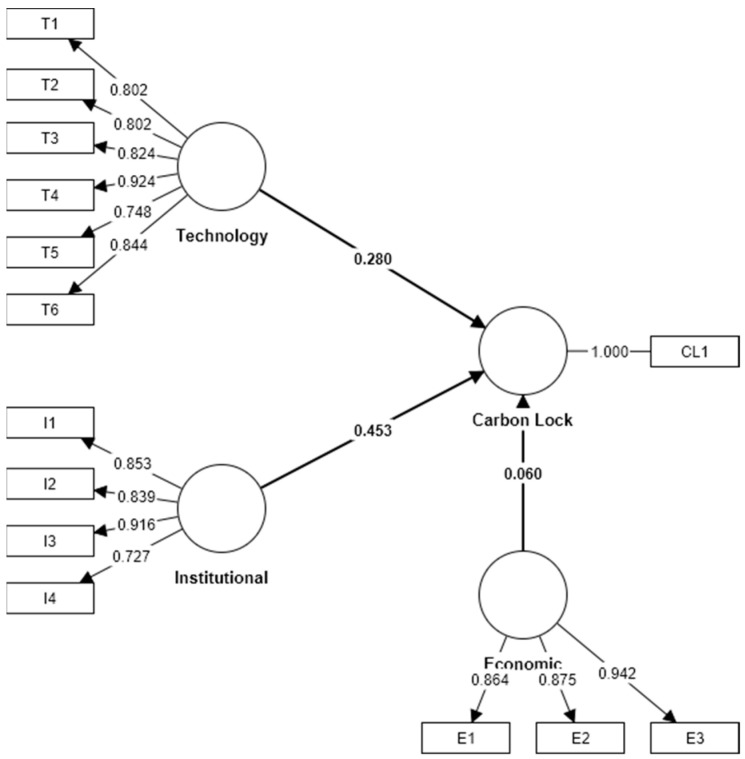
Results of the calculations.

Further calculations were carried out using the evaluated measurement model. The missing values were processed using the PLS algorithm in the SmartPLS v4.0 software. The structural model is shown in Figure 2 and result is shown in Figure 3.

The calculations show that institutional and technical factors are important for the achievement of carbon unlocking targets, while economic factors are less influential (see Table 8). This is also in line with the current research perception that the factors of carbon lock-in mainly originate from technology–institutional lock-in (TIC). Therefore, the achievement of carbon unlocking needs to be approached from both the technical and institutional levels.

However, in practice, certain factors, although having a small impact on the achievement of the target, will not achieve the set target if the role of that factor is missing. Therefore, although the role of economic factors is small, we cannot assume that they will play a small role in achieving the carbon unlock. Therefore, in the next section, we will analyze the necessity of each factor in achieving carbon unlocking and explore which factors are necessary in achieving carbon unlocking.

## 5. NCA Result

**Figure 4:**
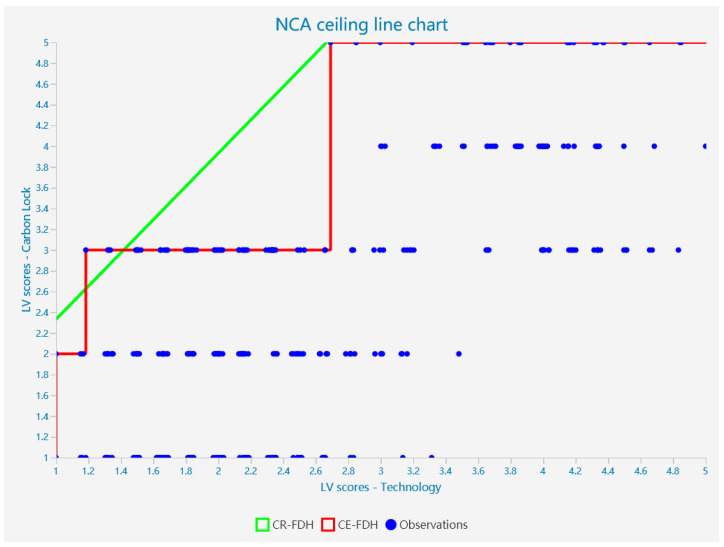
Scatterplot of the analysis of the necessity of technical factors for carbon unlocking.

**Figure 5:**
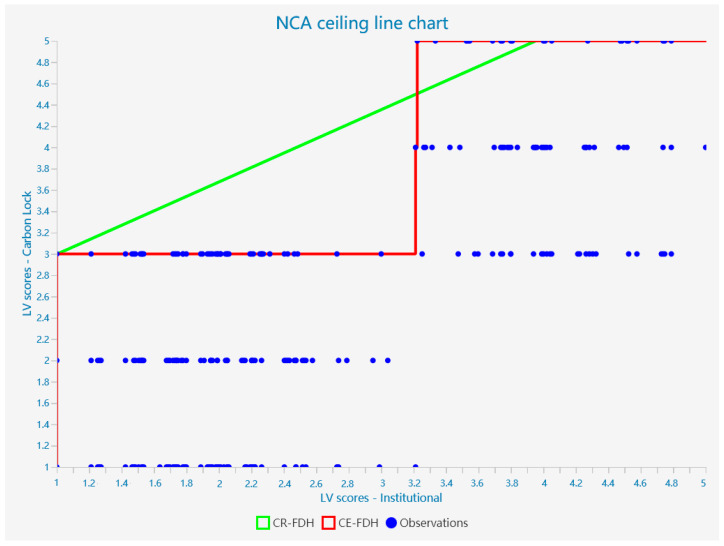
Scatterplot of the analysis of the necessity of institutional factors for carbon unlocking.

**Figure 6:**
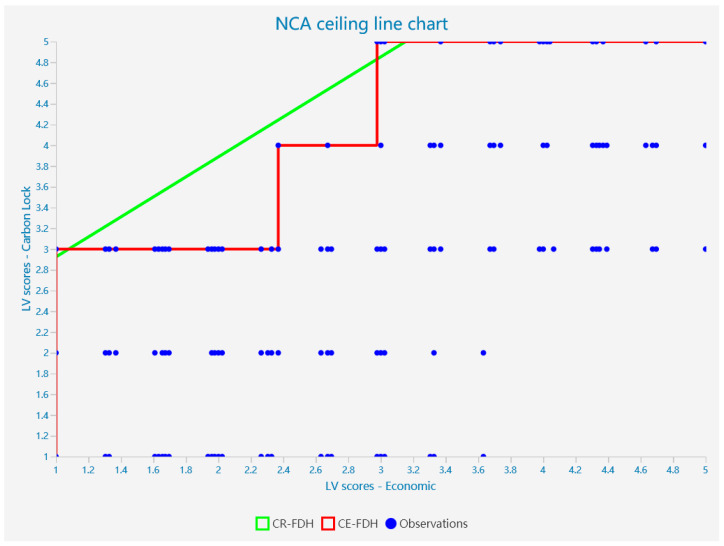
Scatterplot of the analysis of the necessity of economic factors for carbon unlocking.

To further explore the direct relationship between technical, institutional, and economic factors and carbon unlocking, we supplemented the PLS-SEM analysis with a Necessary Conditions Analysis (NCA). Based on the content of the Richter, Alexandre study, we used non-standardized latent variable scores obtained from the PLS-SEM analysis as input for the NCA analysis. We imported them into Smartpls and carried out further analysis. Because of the discrete and hierarchical nature of the data, we selected the CR-FDH for analysis based on existing studies. The graph allows us to further analyze the extent to which each factor limits the achievement of carbon unlocking targets. The scatter plot for the analysis of the necessity of each factor for carbon unlocking is shown in Figure 4, Figure 5 and Figure 6.

### 5.1. Effect Size and Significance Testing

We first calculated and analyzed the values of effect size, accuracy, and slope for the potential variables, as shown in Table 9.

According to Dul, in the NCA analysis, 0 < d < 0.1 is a small effect, 0.1 ≤ d < 0.3 is a medium effect, 0.3 ≤ d < 0.5 is a large effect, and d ≥ 0.5 is a very large effect [10]. Thus, we can find that technology, institutions, and economics all have a moderate effect on the achievement of carbon unlocking in the NCA analysis. At the same time, the reliability of the model meets the requirements. This suggests that technology, institutions, and economics are necessary for the achievement of carbon unlocking, showing a critical medium effect size; the findings are statistically significant (*p* < 0.01).

### 5.2. Bottleneck Analysis

Next, to obtain more detailed conclusions, a bottleneck analysis was carried out (see Table 10). Table 10 shows the minimum values required to achieve the outcome variables for the following predictor variables. At the same time, we can see that only a technical breakthrough is needed to achieve a low level of carbon unlocking. However, a combination of institutional and economic factors is required to achieve a medium to high level of carbon unlocking (>50%). In addition, based on the data in the table, it can be seen that when full unlocking is achieved, the need for economic and institutional factors is higher than the need for technology. This means that full carbon unlocking of transport infrastructure cannot be achieved when a high level of economic and institutional unlocking is not achieved. Carbon unlocking of transport infrastructure needs to be achieved through a combination of economic, institutional, and technological factors. This also means that although economic factors are not a sufficient condition for achieving carbon unlocking, they still play a key role in the process of achieving the goal of carbon unlocking in the transport infrastructure.

## 6. Discussion

This study aims to provide policymakers, transport infrastructure-related enterprises, and others with an updated tool and perspective to assess the influencing factors and methods of achieving carbon unlocking in transport infrastructure, promote the achievement of carbon unlocking targets in transport infrastructure, and facilitate the achievement of carbon neutrality and SDGs targets. This study builds on existing research and combines the PLS-SEM method with the NCA method in order to explore the necessary and sufficient conditions for the achievement of transport infrastructure carbon unlocking targets; the method provides a better understanding of the relationship between variables and outcomes and can explore the demand for each factor if different levels of targets are to be achieved. Therefore, we believe that this study has two key contributions and conclusions as follows.

(1) This study expands the traditional view of the factors influencing carbon lock-in in a theoretical sense. It analyzes the linkages between technical, institutional, and economic factors and carbon unlocking on the basis of necessity and adequacy, and takes into account the need for various indicators to achieve different levels of carbon unlocking. More specifically, the key role of economic factors in achieving carbon unlocking targets for transport infrastructure has been identified. In existing studies, the source of carbon lock-in is usually considered to be technology–institutional lock-in (TIC), and similar results are obtained in this paper when only a sufficient study is done. When analyzed using only PLS-SEM, we can find that the cracking of technical and institutional factors is a key factor in achieving carbon unlocking, with economic factors having minimal influence. However, the adequacy factor only identifies the key conditions for achieving the target, and it is easy to overlook indicators that have a smaller impact but play a key role in achieving the target. Therefore, we added the NCA to this basis and found through the results of the analysis that economic factors have a key role in achieving the carbon unlocking of transport infrastructure. At the same time, according to the calculation results of the bottleneck table, it can be found that to achieve a medium to high degree of carbon unlocking, the combination of three major factors, namely, technology, institutions, and economy, must be achieved, although at the adequacy level, economic factors have less influence on carbon unlocking (0.060). However, if we analyze at the level of necessity, we can find that if we want to achieve a medium to high level of carbon unlocking (>50%), we need a combination of technological, institutional, and economic factors. At the same time, we find that to achieve full carbon unlocking, the requirements for the economic level (0.63) and the institutional level (0.791) are higher than the requirements for the technical level (0.533). In other words, we have achieved an extension of the existing TIC theory and scientifically and rationally argued for the critical role of economic factors in the carbon unlocking process.

(2) This study achieves an expansion of the application level of the method. Although there is a wide range of papers using PLS-SEM for analysis, PLS-SEM is still an emerging method in the transportation field, while the application of NCA in the transportation field, especially in exploring the influencing factors of carbon unlocking in transportation infrastructure, has yet to be further explored [10,84,85]. In this study, we combine PLS-SEM with NCA to explore how the three major elements—technical, institutional, and economic—are integrated into advancing carbon unlocking. We also build on existing research to explore how these two approaches can complement each other in the area of carbon unlocking in transport infrastructure. The second contribution of this paper is therefore to combine PLS-SEM and NCA in the area of transport infrastructure carbon unlocking, identifying the factors that have ‘must’ attributes. The findings can help policy makers and business executives to develop more rational carbon unlocking measures and pathways.

There are several limitations to this study. First, there are many other factors that can influence carbon unlocking that were not included in the survey during the study design process. Second, although we expanded the survey population as much as possible, the sample is still small for the transportation infrastructure sector, and our research and survey were conducted in China; the results may not apply to all countries and regions, as different countries have different economic systems and development models. Therefore, there may be different carbon unlocking pathways in different countries. Third, because policies related to carbon unlocking are still being updated, the results we obtained may only reflect the situation when there are no significant changes in external conditions.

In future studies, we plan to further expand the scope of our investigation and try to use more objective data on the key factors of carbon unlocking in transportation infrastructure. At the same time, we will further supplement and improve the questionnaire in future studies by using more dimensions to describe the institutional, technical, and economic dimensions. In addition, we will further improve the presentation of the questionnaire.

Regardless of the results, however, we offer a new perspective for observing and exploring the factors influencing carbon unlocking. A combination of different approaches may not reveal all carbon unlocking pathways, but the findings of this study still provide value for the realization of carbon unlocking in transport infrastructure. In general, only technical facilitation and support are required to achieve a low level of initial unlocking, but a combination of technical, institutional, and economic factors is required to achieve a medium to high level of unlocking.

Therefore, some recommendations in this article to nurture and coalesce the drivers of transportation infrastructure unlocking are as follows. They include considering internal unlocking power and external unlocking drivers. The external unlocking drivers are provided by the economic policy environment, institutional system environment, and public awareness environment, which are manifested in three aspects. First is the adjustment of national macroeconomic and regional policies. National macro policy changes affect regional production factor flow, allocation, and industrial development orientation, and then impact the locking structure brought by path dependence and incremental scale. Second is the optimization of the performance appraisal system. In the government’s performance appraisal system, the weight of constraint indicators, such as resource consumption, environmental protection, eco-efficiency, and carbon intensity, needs to be strengthened, while ensuring that the government’s commitment is matched with sufficient financial and technical support to ensure that grassroots projects can succeed. Third, public awareness of environmental protection should be raised. The push-back mechanism of climate change requires the public and the government to re-examine the “environmental effects” of energy use, triggering the public’s demand for green development. The general public needs to be convinced that while there might be higher costs for (renewable) energy in the short term, this will quickly guarantee cost savings and an array of other benefits to the whole society, especially in the long term. The internal unlocking momentum comes mainly from the application and diffusion of low-carbon technologies. It is crucial to strengthen technological innovation and implement technological substitution. With the development and maturity of energy-efficient technologies, renewable energy, and other low-carbon technologies, the cost of low-carbon technology substitution is further reduced, changing the balance of high-carbon technologies in the region.

## Figures and Tables

**Table 1:** Key references with approaches.

Dimension	Supporting/Critical Research	Methodology	Weakness	Representative
Technical	Lock-in occurs through combined interactions among technological systems and governing institutions	Narrative exploration	Lack of data support	Unruh [13,14,17]
	The current technologies that have to be replaced	Semi-structured interviews	1. The fairly small set of interviews gives a partial image at a particular time 2. Repetition of the interviews	Janipour [19]
	Path dependencies and increasing returns serve to reinforce existing carbon-intensive transport modes	Cross-case analysis	1. Limited amount of triangulation 2. Possibility of selection bias	Patrick [28]
	Technological change and innovation motivate societies towards greater sustainability	Descriptive analysis	Lack of data analysis	Foxon [23]
	More-granular technologies are empirically associated with faster diffusion, more opportunities to escape lock-in, and higher social returns on innovation investment	Bivariate analysis using diverse samples of technologies	Narrow samples in particular fields of application	Wilson [32]
	Emerging transportation technologies have the potential to surmount carbon lock-in and enable a transition to environmentally sustainable mobility	Nonlinear least squares regression	A selection bias favoring successful diffusion processes	Benjamin [33]
Institutional	Crucial drivers—firm level interactions with technological change, industry leadership, and market structure; government intervention and policy momentum, and citizen involvement and behavior patterns—have played a prominent role in transformations	Historical analysis	1. Is not capable of asserting information regarding the strength of associations between and among events, or between events and other factors 2. No primary data or information	Carley [7]
	Implementation of mandatory energy efficiency building codes can curb rising GHG emissions and reduce the carbon ‘lock-in’ risk	Literature research		Zaid [5]
	Weaken the nature of actor dynamics influencing energy policy, as well as the formal and informal institutions that drive CFPP investments to dislodge carbon lock-in	Empirical analysis	Did not rank the relative importance of individual sources of coal lock-on	Trencher [37]
	Behavioral transformations, institutional shifts, and action by a broad network of actors are key breakout factors to begin decarbonizing economy	Semi-structured interviews	1. No interviews in industries integral to carbon mitigation (such as construction and waste management) 2. Ambiguity surrounding data sources	Susskind [40]
Economic	Industrial structure adjustment leads to carbon emission reduction since it encourages industrial innovation and resource efficiency	An integrated evaluation model based on Input-Output Analysis and Social Network Analysis	More region-specific studies should be undertaken	Li [54]
	Industrial restructuring is considered to be an effective way to achieve economic development and emission reduction	Panel threshold models		Zhou [57]
	There is a role for investment in R&D in addressing climate change	A non-parametric panel data model	R&D intensity did not speak to the development of specific technologies or investment in R&D across sectors.	Awaworyi [65]
	A low-carbon finance index that may help entice foreign direct and private investment in low-carbon energy sector	The DEA-like equation model	Combine multi-dimensional indicators, but usually have no common units of measurement	Mohsin [71]

**Table 2:** Structure of the surveyed population.

Years of Experience	Number	Type of Work	Number
<1	37	Government staff	23
2~5	110	Transport Infrastructure Investors	43
5~10	149	Transport infrastructure construction and operation staff	151
>10	70	Researchers	126
		Others	23

**Table 3:** Factors and indicators influencing carbon unlocking in transport infrastructure.

Factor	Indicator	Item
Technology	The role of technology substitution	What extent do you agree with the statement “the lower the degree of substitution of low-carbon technologies, the more likely they are to lead to carbon unlocking”
	Low-carbon technology diffusion	What extent do you agree with the statement “the less diffusion of low-carbon technologies in the application area, the more likely it is to lead to carbon unlocking”
	Technology R&D path dependency	What extent do you agree with the statement “the more difficult it is to break the existing technology development pathway, the more likely it is to lead to carbon unlocking”
	Difficulty in trading low-carbon technologies	What extent do you agree with the statement “the more difficult it is to trade low-carbon technologies, the more likely it is to lead to carbon unlocking”
	Energy Internet applications	What extent do you agree with the statement “the application of the Internet of Energy in transportation infrastructure has an impact on the degree of carbon unlocking”
	Digital regulatory technologies	What extent do you agree with the statement “the application of digital regulatory technologies in transportation infrastructure has an impact on carbon unlocking control”
Institution	Degree of collusion between government and business	What extent do you agree with the statement “the degree of collusion between government and business in the transportation infrastructure sector has an impact on carbon unlocking”
	Strength of institutional implementation	What extent do you agree with the statement “the strength of the implementation of the relevant system has an impact on carbon unlocking”
	Adaptability of policy development	What extent do you agree with the statement “the degree of carbon locking is influenced by the development of carbon unlocking policies”
	Financial subsidies	What extent do you agree with the statement “the degree of carbon unlocking is influenced by financial subsidies for low-carbon behavior”
	Digital control models	What extent do you agree with the statement “the digital control model in the field of carbon emissions has an impact on carbon unlocking”
Economic	Industrial restructuring	What extent do you agree with the statement “industrial restructuring has an impact on carbon unlocking in transportation infrastructure”
	Share of R&D in low-carbon technologies	What extent do you agree with the statement “the proportion of R&D expenditure on low-carbon technologies has an impact on the degree of carbon unlocking”
	Linkages between low-carbon development patterns and regional economic growth	What extent do you agree with the statement “the degree of influence between low-carbon development patterns and regional economic growth has an impact on carbon unlocking”

**Table 4:** Indicator means and standard deviation.

Latent Variable	Explicit Variable Indicators	Mean	Standard Deviation
Technology	T1	2.615	1.177
	T2	2.552	1.224
	T3	2.634	1.234
	T4	2.473	1.303
	T5	2.697	1.140
	T6	2.555	1.222
Institution	I1	2.443	1.314
	I2	2.549	1.235
	I3	2.459	1.352
	I4	2.751	1.190
Economic	E1	2.661	1.190
	E2	2.631	1.216
	E3	2.511	1.316
Carbon Lock	CL	2.489	1.263

**Table 5:** Indicator means and the loadings of the latent composites.

Latent Variable	Composite Reliability (rho_a)	AVE	Cronbach’s α	Factor Loading	VIF	Explicit Variable Indicators
Technology	0.928	0.682	0.906	0.802	2.323	T1
				0.802	2.383	T2
				0.824	2.722	T3
				0.924	5.587	T4
				0.748	2.445	T5
				0.844	3.252	T6
Institution	0.902	0.700	0.855	0.853	2.161	I1
				0.839	2.290	I2
				0.916	3.233	I3
				0.727	1.523	I4
Economic	0.899	0.800	0.875	0.864	2.307	E1
				0.875	2.282	E2
				0.942	3.302	E3

**Table 6:** Goodness-of-fit.

Indicator	Value	Judgment Criteria
SRMR	0.065	<0.08
d_ULS	0.438	>0.198
d_G	0.292	>0.122
Chi-square	596.971	-
GOF	0.607	>0.26

**Table 7:** Heterotrait–monotrait ratio.

	Carbon Lock	Economic	Institution	Technology
Carbon Lock				
Economic	0.63			
Institution	0.78	0.85		
Technology	0.73	0.79	0.93	

**Table 8:** Results from the structural model.

Direct Effects	Path Coefficient	t	*p*	CI	
				2.5%	97.5%
Economic -> Carbon Lock	0.06	1.148	0.251	−0.044	0.167
Institutional -> Carbon Lock	0.453	6.988	<0.01	0.323	0.58
Technology -> Carbon Lock	0.28	4.457	<0.01	0.155	0.404

**Table 9:** Results of the necessity analysis.

Latent Variable	Effect Size (d)	Accuracy	Slope	Intercept	Condition Inefficiency	Outcome Inefficiency	*p* Value
Economic	0.14	97.814	0.964	1.961	46.192	48.121	<0.01
Institutional	0.185	96.175	0.678	2.319	26.184	49.941	<0.01
Technology	0.139	98.907	1.602	0.731	58.366	33.317	<0.01

**Table 10:** Bottleneck analysis table.

Degree of Carbon Unlocking	Carbon Lock	Economic	Institutional	Technology
0%	1	n/a	n/a	n/a
10%	1.4	n/a	n/a	n/a
20%	1.8	n/a	n/a	n/a
30%	2.2	n/a	n/a	n/a
40%	2.6	n/a	n/a	1.167
50%	3	1.078	1.003	1.417
60%	3.4	1.493	1.593	1.666
70%	3.8	1.908	2.183	1.916
80%	4.2	2.323	2.773	2.166
90%	4.6	2.737	3.363	2.416
100%	5	3.152	3.953	2.665

## Data Availability

The raw data supporting the conclusions of this article will be made available by the authors without undue reservation.

## Abbreviations

AVE	Average Variance Extracted
CB-SEM	Covariance Base-Structural Equation Modeling
CR	Composite Reliability
CR-FDH	Ceiling Regression-Free Disposal Hull
HTMT	Heterotrait–Monotrait ratio
NCA	Necessary Condition Analysis
PLS-SEM	Partial Least Squares Structural Equation Modeling
R&D	Research and Development
SDGs	Sustainable Development Goals
TIC	Techno-Institutional Complex
VIF	Variance Inflation Factor
SRMR	Standardized Root Mean Square Residual
